# Perirectal Mucinous Adenocarcinoma After Subtotal-Colectomy for Crohn’s Disease: A Case Report

**DOI:** 10.7759/cureus.55305

**Published:** 2024-03-01

**Authors:** Matthew S Wishnoff, Ashley Shustak, Stephen P Sharp

**Affiliations:** 1 General Surgery, Virginia Commonwealth University (VCU) Health System, Richmond, USA; 2 Colon and Rectal Surgery, Virginia Commonwealth University (VCU) Health System, Richmond, USA

**Keywords:** subtotal abdominal colectomy, colorectal cancer recurrence, proctocolectomy, crohn’s disease, mucinous adenocarcinoma

## Abstract

Colorectal carcinoma (CRC) represents the third most common cancer and the second highest cause of cancer-related death in the United States. CRC is particularly prevalent in patients with underlying inflammatory bowel disease. Adenocarcinoma represents more than 90% of new CRC diagnoses. The mucinous subtype of colorectal adenocarcinoma is found in approximately 10-20% of all colorectal cancer patients and is most frequently located in the proximal colon. We report a case of mucinous adenocarcinoma arising from the rectal stump of a patient who had previously undergone subtotal-colectomy with end ileostomy for Crohn’s disease. She initially presented with gradually worsening chronic abdominal pain and gelatinous rectal discharge. She was found to have a complex cystic lesion communicating with her Hartman's pouch. She ultimately underwent a completion proctectomy, radical hysterectomy, and bilateral salpingo-oophorectomy in conjunction with gynecology oncology. To the best of our knowledge, this case represents the first description of a perirectal mucinous adenocarcinoma arising in a patient after subtotal-colectomy for Crohn's disease.

## Introduction

Colorectal carcinoma (CRC) represents the third most common cancer and second most cause of cancer-related death in the United States. Patients with inflammatory bowel disease (IBD) such as Crohn’s disease and ulcerative colitis are six times more likely to develop CRC as compared to the general population. The presence of IBD with colonic involvement is one of the top three risk factors for CRC development. Adenocarcinoma is the most identified colorectal neoplasm and represents more than 90% of all identified cancers. The mucinous subtype accounts for 10-20% of all colorectal adenocarcinomas and is most frequently located in the proximal colon [1]. One of the most cited mechanisms for CRC development in patients with IBD is chronic bowel inflammation leading to dysplasia and eventually cancer [2]. Several case-control studies have found a significant correlation between the severity of colonic inflammation and the risk of neoplastic changes [3]. The finding of high-grade dysplasia should prompt referral for total proctocolectomy in patients with IBD due to the high prevalence of concurrent malignancy elsewhere in the colon [4]. 

## Case presentation

A 62-year-old female with long-standing Crohn’s disease and a history of multiple abdominal surgeries leading to a subtotal-colectomy and end ileostomy in her twenties presents with gradually worsening chronic abdominal pain and one week of gelatinous rectal discharge. She endorsed anorexia but the review of systems was otherwise negative. 

**Figure 1:**
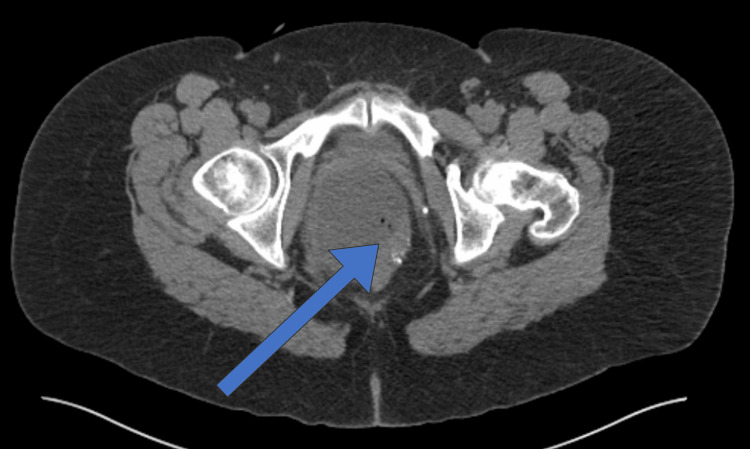
CT abdomen/pelvis with an arrow demonstrating pelvic fluid-filled cavity communicating with the rectal stump and containing locules of gas and scattered calcifications

Physical exam at the time of presentation was significant for mild tenderness in the upper abdomen, healthy appearing ileostomy with stool output, and mucinous drainage from the rectum. The digital rectal exam was aborted due to pain, however, there was no external induration or erythema. CT scan was significant for an 8 cm x 12 cm pelvic fluid collection with some air and calcification present between the rectum and vagina (Figure 1).

**Figure 2:**
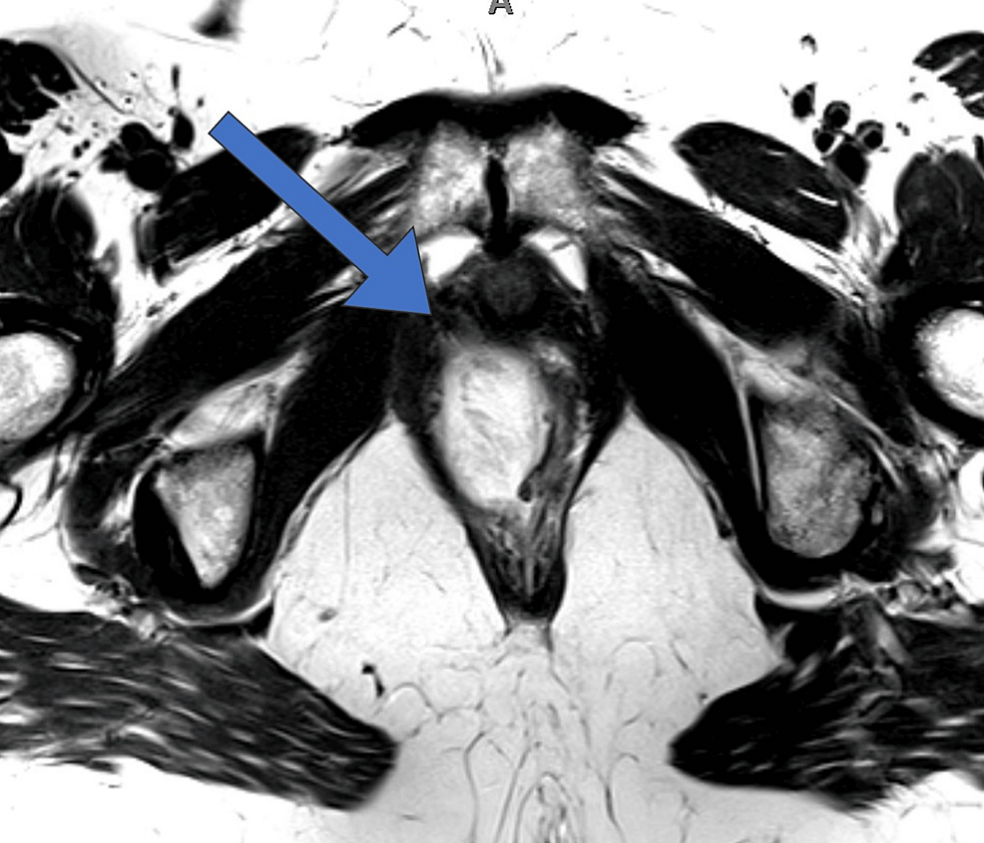
MRI demonstrating a pelvic mass with an arrow demonstrating communication to the lower rectal lumen at the 10:00 - 12:00 position

Hematology revealed a leukocytosis of 12,000, and blood culture obtained at the time of admission would later reveal group B streptococcus. MRI revealed a complex cystic lesion in the right pelvis paralleling rectal Hartman's pouch, communicating with the lower rectal lumen via a small rectal wall defect, as well as centripetal frond-like enhancement emanating from the wall of the lower half of the lesion concerning for possible mucinous neoplastic lesion (Figure 2).

The collection was biopsied via fine needle aspiration, which revealed predominately mucous and few histiocytes, but no malignant cells were identified. Sigmoidoscopy confirmed the presence of a large fistulous communication between the rectum and a cystic cavity. She was concurrently evaluated by gynecologic-oncology who were concerned that this could represent an ovarian neoplasm. 

The case was discussed at our multidisciplinary conference and the decision was made to proceed with a joint robotic assisted completion abdominoperineal resection and radical hysterectomy with bilateral salpingo-oophorectomy. Upon entering the pelvis, the mass was densely adhered to all pelvic structures including the posterior wall of the vagina, and bilateral pelvic sidewalls, and inseparable from the rectal stump. The entirety of the mass and associated structures were removed en block without collateral damage. Final pathology revealed adenocarcinoma arising in the rectum with fistula tract and secondary involvement of the right ovary and portion of the right fallopian tube. Histologic sections of the pelvic mass show an adenocarcinoma with abundant extracellular mucin. Immunohistochemical stains performed show that the tumor was positive for CK7, CK20, and CDX2 negative for PAX 8. 

## Discussion

Colon and rectal cancers are the second leading cause of cancer-related deaths and represent the third most common cancer in the United States. Both genetic and environmental factors contribute to the development of CRC. Although several genetic disorders impart a substantial risk of cancer, most CRCs are sporadic. Normal colonic epithelium undergoes dysplastic changes to form a precancerous lesion (adenoma) and then further accumulates genetic mutations to give rise to invasive carcinoma. Adenocarcinoma represents more than 90% of new CRC diagnoses [5]. The mucinous subtype of colorectal adenocarcinoma is found in approximately 10-20% of all colorectal cancer patients and is most frequently located in the proximal colon [6]. Histologically it is defined by >50% of the tumor volume being composed of extracellular mucin [7]. 

There is a well-documented association between inflammatory bowel disease (IBD) such as Crohn’s disease and ulcerative colitis and the development of CRC. The increased prevalence of CRC in IBD is due to the chronic inflammation-induced dysplasia-carcinoma sequence. As expected, risk factors for CRC development in CD include younger age at CD diagnosis and disease duration > 10 years. While non-mucinous adenocarcinoma is still the most prevalent CRC identified in patients with Crohn’s disease, there is a higher prevalence of the mucinous subtype in patients with Crohn's disease as compared to the general population [8] Compared to non-mucinous adenocarcinoma, patients with the mucinous subtype are usually diagnosed at a more advanced stage and have a poorer response to chemotherapies. A recent population-based study reported an approximately 93% 5-year survival rate for patients with localized disease [9]. 

There are a few reports of mucinous adenocarcinoma occurring within fistula tracks in patients with Crohn’s disease. These cases occur most often in the setting of recurrent peri-anal and peri-rectal fistulous disease [7,10]. Even more rare is the presence of mucinous adenocarcinoma after proctocolectomy. In fact, there has only been one case report of a mucinous adenocarcinoma arising in a patient after proctocolectomy for Crohn’s disease, which was a report of a 50-year-old female presenting with a perineal mucinous adenocarcinoma eleven years after a proctocolectomy. This was treated via exenteration of the vagina, uterus, ovaries, and coccygeal bone [11]. To our knowledge, this is the first description of a perirectal mucinous adenocarcinoma arising in a patient after subtotal-colectomy for Crohn's disease.

## Conclusions

Numerous studies have shown that colonoscopic surveillance in patients with inflammatory bowel disease (IBD) is associated with a reduction in the prevalence of colorectal carcinoma development and mortality. The standard treatment for low rectal adenocarcinoma is abdominoperineal resection with local extension into surrounding structures to ensure en bloc removal of the malignancy, which was the ultimate treatment strategy for our patient. However, prevention remains a core tenant of colorectal cancer care. The finding of high high-grade dysplasia should prompt referral for total proctocolectomy in patients with IBD due to the high prevalence of concurrent malignancy elsewhere in the colon. Overall, these principles and this case presentation suggest continued surveillance in patients with residual intestinal tissue after subtotal colectomy. 

## References

[1] Thrumurthy SG, Thrumurthy SS, Gilbert CE, Ross P, Haji A (2016). Colorectal adenocarcinoma: risks, prevention and diagnosis. BMJ.

[2] Muthusami S, Ramachandran IK, Babu KN (2021). Role of inflammation in the development of colorectal cancer. Endocr Metab Immune Disord Drug Targets.

[3] Mattar MC, Lough D, Pishvaian MJ, Charabaty A (2011). Current management of inflammatory bowel disease and colorectal cancer. Gastrointest Cancer Res.

[4] Zisman TL, Rubin DT (2008). Colorectal cancer and dysplasia in inflammatory bowel disease. World J Gastroenterol.

[5] Huang A, Yang Y, Shi JY, Li YK, Xu JX, Cheng Y, Gu J (2021). Mucinous adenocarcinoma: a unique clinicopathological subtype in colorectal cancer. World J Gastrointest Surg.

[6] Luo C, Cen S, Ding G, Wu W (2019). Mucinous colorectal adenocarcinoma: clinical pathology and treatment options. Cancer Commun (Lond).

[7] Freeman HJ, Perry T, Webber DL, Chang SD, Loh MY (2010). Mucinous carcinoma in Crohn's disease originating in a fistulous tract. World J Gastrointest Oncol.

[8] Ishimaru K, Tominaga T, Nonaka T (2021). Colorectal cancer in Crohn's disease: a series of 6 cases. Surg Case Rep.

[9] Xie GD, Liu YR, Jiang YZ, Shao ZM (2018). Epidemiology and survival outcomes of mucinous adenocarcinomas: a SEER population-based study. Sci Rep.

[10] Smith R, Hicks D, Tomljanovich PI, Lele SB, Rajput A, Dunn KB (2008). Adenocarcinoma arising from chronic perianal Crohn's disease: case report and review of the literature. Am Surg.

[11] Keese M, Back W, Dinter D, Gladisch R, Joos A, Palma P (2005). Case report: late perianal mucinous adenocarcinoma after Crohn's disease proctectomy: an oncological rarity. World J Surg Oncol.

